# Aphid Parasitoid Mothers Don't Always Know Best through the Whole Host Selection Process

**DOI:** 10.1371/journal.pone.0135661

**Published:** 2015-08-13

**Authors:** Quentin Chesnais, Arnaud Ameline, Géraldine Doury, Vincent Le Roux, Aude Couty

**Affiliations:** FRE CNRS 3498 EDYSAN (Écologie et Dynamique des Systèmes Anthropisés), Université de Picardie Jules Verne, 33 rue St Leu, F-80039, Amiens Cedex, France; University of Natural Resources and Life Sciences, Vienna, AUSTRIA

## Abstract

Parasitoid host selection behaviour has been extensively studied in experimentally simplified tritrophic systems formed by one single food chain (one plant, one herbivore and one parasitoid species). The "Mother knows best" hypothesis predicts that the preference for a plant-host complex should be positively correlated with plant quality for offspring performance. We studied the host selection behaviour of the generalist endoparasitoid *Aphidius matricariae* towards the black bean aphid *Aphis fabae* in the intercrop system including *Vicia faba* as a focal plant and its companion plant *Camelina sativa*. Dual-choice laboratory bioassays revealed that parasitoid females preferred to orientate towards (1) the plant-aphid complex over the non-infested plant whatever the complex (2) the *C*. *sativa-A*. *fabae* complex over the *V*. *faba-A*. *fabae* complex. In dual choice attack rate bioassays, parasitoid females showed more interest towards the aphids on *C*. *sativa* but paradoxically chose to oviposit more in aphids on *V*. *faba*. Ultimately, parasitoids that had developed on the *V*. *faba-A*. *fabae* complex exhibited better fitness parameters. By demonstrating that parasitoid females were able to discriminate the aphid host that offered the highest fitness to their offspring but selected beforehand the least suitable plant-aphid complex, we provide key insight into the disruption in their host selection behaviour potentially triggered by diverse habitats. This suggests that the "Mother knows best" hypothesis could be thwarted by increasing the complexity of the studied systems.

## Introduction

The "Mother knows best" hypothesis, also known as the "preference-performance hypothesis", derives from the general optimality theory originally set for phytophagous insects [1–4], which states that female oviposition preferences should positively correlate with host suitability for offspring development (*i*.*e*. offspring survival and further adult fecundity). The "Mother knows best" hypothesis has been recently expanded to parasitoid insects [5], natural enemies of phytophagous insects. Because their larvae develop as obligatory parasites, the reproductive success of parasitoids is partly determined by the ability of females to select a suitable insect host for the development of their progeny. Physical and chemical cues associated with the insect host and/or its habitat have been shown to play important roles in the localization and selection of both the insect host and its host plant (for review, see [6–11]). During the first steps of host habitat and host location, parasitoid females may respond to volatile chemical blends produced by their host plant, to a combination of insect host and host plant odours and/or to volatiles produced by the plant in response to host feeding damage (*i*.*e*., herbivore induced-plant volatiles or HIPV). Once the parasitoid female has reached a potential insect host habitat, it begins to search for the insect host on or near the host plant and responds to chemical stimuli produced by the insect host itself or arising from its products rather than to host-plant derived products. The ability of koinobiont parasitoid (*i*.*e*. parasitoids whose hosts continue to feed and grow after parasitization) females to reliably predict future host quality has been demonstrated in choice studies where the preferred insect hosts were indeed the ones that allowed maximum adult parasitoid size and/or minimum development duration [12,13]. However, although it is also expected that the preferred host plant and/or plant-insect host complex will provide an advantage in terms of fitness for the parasitoid offspring, there is a lack of empirical evidence to link the orientation preferences of parasitoid females during the early steps of host selection with offspring performance [14]. Instead, most experiments have ignored the role played by the first trophic level (*i*.*e*. the plant) and focused on preference and performance by parasitoids in a bitrophic system (for review, see [8,15]). To fill this lack of knowledge, the "Mother knows best" hypothesis should be considered, for parasitoid insects, not only through the study of the entire host selection process (from the early stages of host habitat selection to the final steps of host acceptance/suitability), but also through a dual choice set up between at least two host habitats. Indeed, laboratory studies generally tend to simplify multitrophic interactions that occur in nature. Parasitoid host selection behaviour has been extensively studied using “simple” tritrophic systems formed by one single food chain that consisted of one plant, one phytophagous host and one parasitoid species [7,16,17]. Such studies often neglect the impact of complex odour bouquets whereas, in natural systems, parasitoids search for their host in habitats that are spatially and temporally diverse, and comprise various plants and herbivore communities [18].

Intercropping systems involve the culture of at least two crops in the same space and time [19] with one focal crop associated with one companion plant providing benefits such as pest/weed control and/or increased yields. They provide an opportunity to test theoretical ecology concepts and study the effects of plant diversity on food web interactions. Thus, they represent interesting models to investigate the links between parasitoid foraging behaviour in diverse habitat and parasitoid performance.

In the present work we used a laboratory approach to test the "Mother knows best" hypothesis through the study of all the steps of the host selection process by an aphid parasitoid facing two plant-host complexes. In other words, will the plant-host complex initially preferred by the parasitoid females be the one consistently preferred along all the steps of the host selection process, and will it ultimately allow the best progeny performance? To address this question, our experimental food web was formed of two crop plants, the broad bean *Vicia faba* (L.) (Fabaceae) as the focal plant and the false flax *Camelina sativa* (L.) Crtz. (Brassicaceae) as the companion plant, the black bean aphid *Aphis fabae* (Homoptera: Aphididae) as the host and its parasitoid wasp *Aphidius matricariae* Haliday (Hymenoptera: Braconidae: Aphidiinae). Three experiments were set, taking into account each of the hierarchical steps occurring along the entire host selection process:

1. The first steps of host selection (host habitat and host location) were studied by assessing the preferences of *A*. *matricariae* females for a host plant (*V*. *faba* or *C*. *sativa*), depending on its status (infested or not by *A*. *fabae*).
2. Host recognition and acceptance of *A*. *fabae* by *A*. *matricariae* females were evaluated on both plants through an attack rate bioassay (dual choice experiment).
3. Host suitability/regulation was assessed by comparing the fitness of the parasitoid offspring that developed on each of the two plant-host complexes.

## Materials and Methods

### Study system

Camelina (*C*. *sativa*) is a Brassicaceae which was an important cultivated oil crop in temperate Europe until the nineteenth century [20]. It has recently been re-introduced because its oil offers good opportunities not only as a biofuel but also as functional food due to its exceptionally high levels of omega-3 fatty acids [21]. Camelina is generally reported to be tolerant and resistant to various pathogens and insects [22,23], although it has recently been shown to be a potential host for some aphid pests [24]. The faba bean (*V*. *faba*) is widely grown under a range of climatic conditions from temperate to subtropical where it hosts a wide variety of insect pests [25]. Camelina can be used as a companion plant in intercropping systems with faba bean, for weed control [26]. The black bean aphid *A*. *fabae* is a polyphagous species with a wide host range including Fabaceae but also Brassicaceae plants [27], favouring host plant alternation. This aphid is one of the most damaging pests of faba bean plants. It causes direct damage by phloem feeding, which results in significant impairments of plant growth and yield [28], and it also acts as a vector for plant viruses [29]. The black bean aphid is a common host of the generalist and cosmopolitan hymenopteran parasitoid wasp *A*. *matricariae* that uses more than a hundred different aphid species as hosts [30] and is commercially available for biological control.

### Laboratory assays

#### Plants and Insects

Plantlets used for the experiments were obtained from seeds. *Camelina sativa* (cv. “Calena”) were provided by Semences de l'Est, Reims (France) and *Vicia faba* (cv.”Espresso”) by the Fédération REgionale de Défense contre les Organismes Nuisibles (FREDON) of Picardie (France). They were grown for three weeks, in plastic pots (60 x 60 x 70 mm) with commercial sterilized potting soil in growth chambers (SANYO, Versatile Environmental Test chamber) under controlled conditions (20 ± 1°C, 60 ± 5% relative humidity, and a 16L:8D photoperiod at 2 klux).

The colony of *Aphis fabae* was initiated from a single apterous parthenogenetic female (provided in 2012 by Gembloux Agro-Bio-Tech, Belgium) and was maintained in ventilated plastic cages (360 x 240 x 110 mm) in growth chambers under controlled conditions (20 ± 1°C, 60 ± 5% relative humidity, and a 16L:8D photoperiod at 2 klux) to induce parthenogenesis. The two plant-aphid combinations used in the study were obtained by mass-rearing of *A*. *fabae* either on *V*. *faba* or *C*. *sativa* plants.

Cohorts of synchronized *A*. *fabae* nymphs were reared on plantlets of each of the two host plants under the controlled conditions described above until they were used for the experiments. They were obtained by a procedure consisting in placing parthenogenetic adult females on plantlets for 24 hours before removing them. Three-day-old aphids (second instar larvae) were randomly selected as hosts for all bioassays.

*Aphidius matricariae* (Haliday) parasitoids (Hymenoptera: Aphidiidae) were obtained from Viridaxis, Gosselie (Belgium) as mummies. Attention was paid to ensure the use of a commercial line of *A*. *matricariae* that would have been reared neither on the aphid *A*. *fabae* nor on any of the plant species used in the study. Upon reception, mummies were transferred to plastic tubes (75 x 13 mm) closed with a cotton plug. Once emerged, parasitoids were sexed and mating was allowed by grouping three to four males with six to seven females in the same tube. They were fed *ad libitum* with a 1:1 honey/water (v/v) solution until used for the experiments. Parasitoids were maintained in a climate room at 20 ± 1°C, 60 ± 5% relative humidity, and a 16L:8D photoperiod. Three-day-old standardized parasitoid females (mated, fed and without oviposition experience) were randomly selected for the laboratory experiments.

### Bioassay 1: Habitat and host plant localization

**Fig 1:**
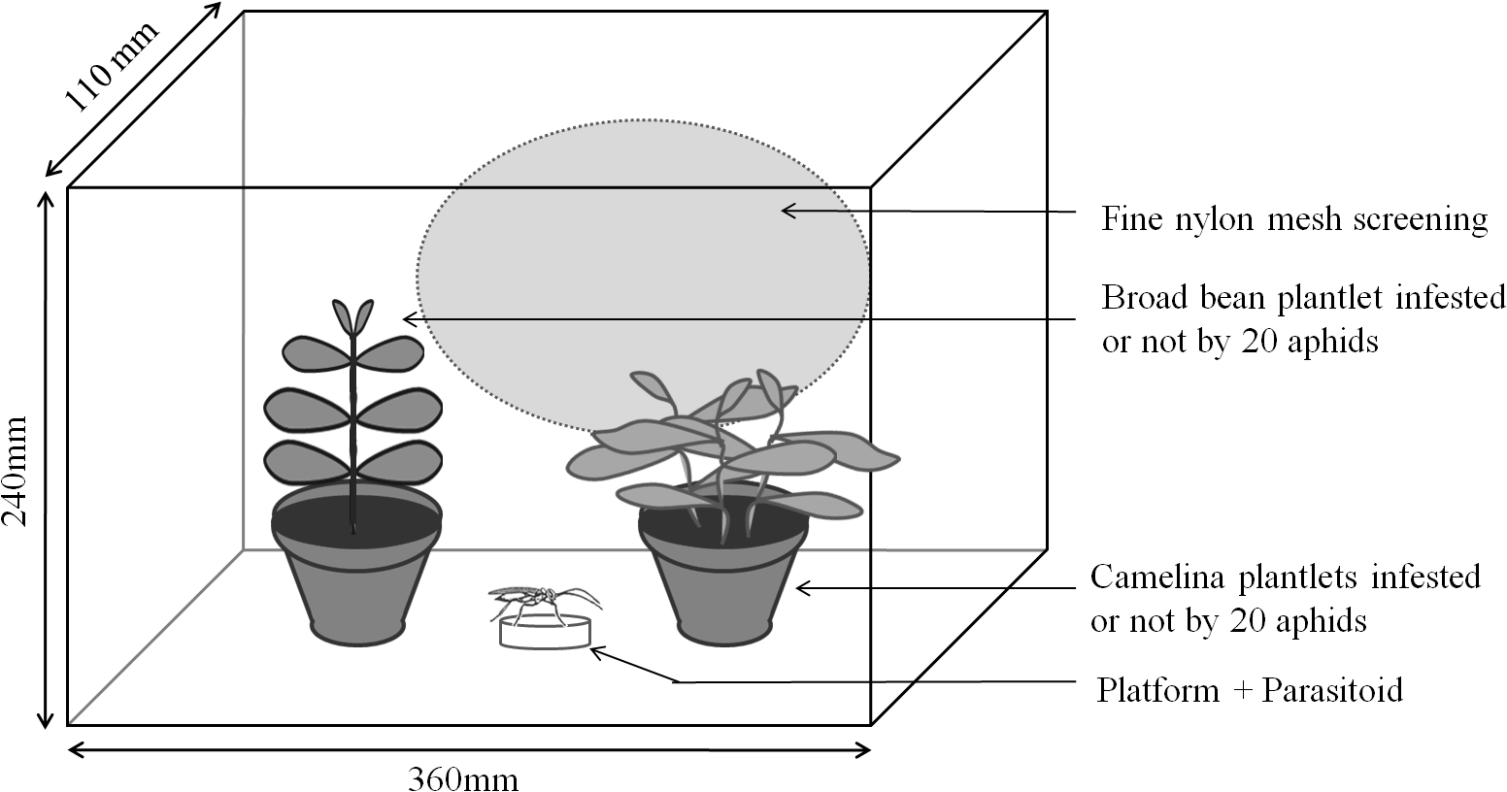
Experimental device used for the preference experiment.

The aim of bioassay 1 was to determine the preference of *A*. *matricariae* females in dual choice tests using different combinations of the two plants. The experimental setup used was modified from [31]. It consisted of four ventilated plastic chambers (360 x 240 x 110 mm) used simultaneously, inside which *V*. *faba* and *C*. *sativa* plants were placed on opposite sides of the chamber (Fig 1). In order to use potted plants of similar biomasses, one pot contained one plantlet of *V*. *faba* whereas the other pot contained three plantlets of *C*. *sativa*. This ensured that aphids were submitted to similar amounts of COVs potentially emitted by each host plant. To limit possible biases from the environment around the chambers, the relative position of the two plants in the ventilated chambers was inverted every replicate. Chambers were randomly positioned within the room where they received homogenous light from above. Four combinations were randomly tested: 1) non-infested *C*. *sativa vs*. non-infested *V*. *faba*; 2) *A*. *fabae*-infested *C*. *sativa vs*. non-infested *V*. *faba*; 3) non-infested *C*. *sativa vs*. *A*. *fabae*-infested *V*. *faba*; 4) *A*. *fabae*-infested *C*. *sativa vs*. *A*. *faba*e-infested *V*. *faba*. An infested plant was obtained by placing 20 *A*. *fabae* neonates on the plantlet for 72 hours prior to the test, to ensure induction of plant responses [32]. A single standardized *A*. *matricariae* female was placed with a small paintbrush on a take-off platform (*i*.*e*. Petri dish lid, 50 mm in diameter) in the centre of the experimental setup. Female parasitoids were continuously observed until they made a first choice (*i*.*e*. landing on either plant) or for a maximum of twenty minutes after introduction. This duration was chosen as preliminary tests showed that 50% of the females responded within 5 minutes. Only females that landed on one of the two host-plant species were considered as responding parasitoids. Times from introduction to first choice by responding females were recorded (latency time). The females that were not found on any plant, *i*.*e*. that were on inner walls or ground of the experimental chamber, were considered as non-responding parasitoids. Females that did not leave the take-off platform within 20 minutes were discarded. Thirty-eight to ninety-one replicates per treatment were performed. All experiments were conducted at 20 ± 1°C and 60 ± 5% relative humidity.

### Bioassay 2: Host recognition and acceptance

The aim of bioassay 2 was to evaluate how *A*. *matricariae* females would locate and evaluate their host after habitat location had been achieved. In a choice test, the attack rate and the success of attack of *A*. *matricariae* females were measured on aphid hosts previously reared on each plant. Attack rate arenas were adapted from [33] and consisted of 90 mm diameter plastic Petri dishes (Gosselin, Hazebrouck, France), containing one leaf of *V*. *faba* and one leaf of *C*. *sativa* embedded in 1.5% agar (Prolabo, Louvain, Belgium) and separated from each other by a distance of 2 cm. Although they were not found significantly different at the 5% level, leaf surfaces slightly differed (*C*. *sativa*: 6.066 ± 0.250 cm²; *V*. *faba*: 6.814 ± 0.268 cm², Mann-Whitney rank-sum test, *U* = 76, P = 0.053). Ten three-day-old *A*. *fabae* were deposited onto each leaf 24 hours prior to the experiment. *A*. *fabae* reared on *V*. *faba* were deposited on *V*. *faba* leaves and *A*. *fabae* reared on *C*. *sativa* were deposited on *C*. *sativa* leaves.

One standardized *A*. *matricariae* female was carefully introduced inside the attack rate arena. Observations started immediately and lasted for 10 min. The time before the first recorded behavioural item (latency time), the first choice (aphid patch which was reached first by the parasitoid) and the second choice were noted. Then, the frequency and the sequence of the following behavioural items were recorded (AE: Antennal Examination, AB: Abdomen Bending and OI: Ovipositor Insertion) [9]. An ovipositor insertion was recorded whenever a parasitoid female made physical contact with an aphid using its ovipositor, while exhibiting an oviposition stance (Abdomen Bending). For the analysis, the behaviours were regarded as a series of events and only the final event was recorded (*e*.*g*. if a wasp had antennated an aphid and then bent its abdomen, this was noted as AB and not as AE). Each contact between a wasp and an aphid was classified in only one of the above categories. Even if the number of the different behavioural items might not be affected, transition frequencies between behaviours may change [34]. Therefore, all behavioural items and their sequential order were recorded and computed into an ethogram. To assess the proportion of Ovipositor Insertion (OI) resulting in true oviposition (OV), all stung aphids were dissected in a drop of NaCl solution (9 ‰) under a stereomicroscope to calculate the rate of oviposition (%_OV = Number of true oviposition (OV) / Number of ovipositor insertion (OI)). Thirty replicates in total were made and all experiments were conducted at 20 ± 1°C and 60 ± 5% relative humidity.

### Bioassay 3: Host suitability

The aim of bioassay 3 was to determine the effects of the plant-aphid complexes, either *C sativa*-*A*. *fabae* or *V*. *faba-A*. *fabae*, on the fitness of the parasitoid progeny.

Preliminary experiments had been conducted in order to evaluate the potential effect of aphid treatment (*i*.*e*. previously reared on *C*. *sativa* or *V*. *faba*) on the probability of laying an egg in each attacked host (probability of true oviposition) under experimental conditions of controlled oviposition. The controlled oviposition procedure consisted of placing a single standardized *A*. *matricariae* female (*i*.*e*., mated, fed, and without oviposition experience) with a single three-day-old *A*. *fabae* nymph in a small Eppendorf tube (0.5 ml), as described in [35]. Each parasitoid female was only used once. For each host plant, 24 *A*. *fabae* coming from each rearing plant were dissected under a stereomicroscope immediately after ovipositor insertion by *A*. *matricariae* to determine the presence or absence of a parasitoid egg. The frequency of true oviposition (%_OV) (87.50% for aphids reared on *V*. *faba* and 83.33% for those reared on *C*. *sativa*) was not significantly affected by the host-plant species on which aphids had been previously reared (Fisher’s exact test, *P* > 0.80).

Prior to the oviposition procedure, each three-day-old *A*. *fabae* nymph was measured under a stereomicroscope (LEICA M165C) from the tip of the head to the base of the cauda. After being stung by a parasitoid female, each aphid nymph was then individually placed back onto its host plant in a clip-cage under the controlled conditions described above. It was followed and observed daily until death or formation of a mummy. In the latter case, it was measured as described above and transferred to a plastic tube (75 x 13 mm) closed with a cotton plug. Emerged parasitoids were sexed and females were fed *ad libitum* with a 1:1 honey/water solution for three days to ensure they had reached their fecundity peak [36]. They were then stored at -80°C for further measurements. The tibia length of females, used as a proxy for parasitoid size, was measured as described above for aphids. Females were dissected into a drop of NaCl solution (9 ‰) to collect their ovaries and the total number of eggs present in the two ovaries was recorded.

The following parasitoids’ life-history parameters were computed: 1) Pre-nymphal developmental time (from oviposition to mummification) in days; 2) Nymphal developmental time (from mummification to adult emergence) in days; 3) Total developmental time (from oviposition to adult emergence) in days; 4) Mummy size (length in mm); 5) Tibia length (in mm) of parasitoid females; 6) Egg load of parasitoid females; 7) Mummification rate (no. of mummies / no. of stung aphids) x 100; 8) Emergence rate (no. of emerged parasitoids / no. of mummies) x 100.

### Statistical analysis

Mean values are given with their standard error of the mean (SEM). Preferences of *A*. *matricariae* females for different combinations of tested plants in Bioassay 1, the first choice in Bioassay 2 and the rate of oviposition were compared using a Chi-square test. The Kruskal-Wallis test was performed to assess the effect of plant combination on the percentage of non-responding parasitoids in Bioassay 1. Parasitoid attack rate parameters (AE, AB, OI and latency time), developmental times (pre-nymphal, nymphal and total) and mummy and tibia lengths were compared using Student *t*-tests for independent samples. Aphid sizes were compared using Student *t*-tests, by a randomized selection of fifty *A*. *fabae* reared on each host plant. Mummification rate, emergence rate and sequential analysis were compared using Fisher’s exact tests. All statistical analyses were carried out using the statistical program ‘R’ version 3.1.0 [37].

## Results

### Bioassay 1: Habitat and host plant localization

**Fig 2:**
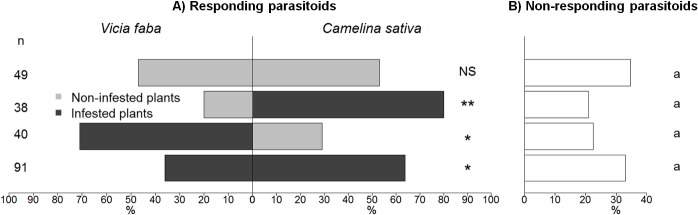
Percentages of responding (A) *Aphidius matricariae* females that landed on each presented plant at the end of the 20-min dual-choice bioassay (grey bars). Different combinations of aphid-infested (dark grey bars) or non-infested (light grey bars) *Vicia faba* and *Camelina sativa* plantlets were presented to female wasps. The percentage of non-responding (B) parasitoids is presented on the right of the chart (white bars). Asterisks indicate significant differences (* *P* < 0.05; ** *P* < 0.01) in the choice made by responding parasitoids (Chi-square test). Same letters indicate non-significant differences (Kruskal-Wallis test, *P* > 0.05) between the percentages of non-responding parasitoids.

When submitted to a dual-choice involving two non-infested plants (Fig 2 A; S1 Table), *A*. *matricariae* females did not exhibit any preference (*χ* ^2^-test, *χ* ^2^ = 0.125, *P* = 0.72). Infested plants were preferred over non-infested plants, whatever the plant-*A*. *fabae* complex (*χ* ^2^-test, *χ* ^2^ = 10.800, *P* = 0.001 for *C*. *sativa*-*A*. *fabae* and *χ* ^2^ = 5.452, *P* = 0.020 for *V*. *faba-A*. *fabae*; S2 and S3 Tables). In the presence of both infested plants, *A*. *matricariae* females exhibited a significant preference for the *C*. *sativa*-*A*. *fabae* complex (*χ* ^2^-test, *χ* ^2^ = 4.737, *P* = 0.029; S4 Table). The percentage of non-responding parasitoids was not significantly affected by the plant combination presented to the female parasitoid (Kruskal-Wallis test, *H* = 3.40, df = 3, *P* = 0.33; Fig 2B).

Latency times (seconds; mean ± SEM) were not significantly different: non-infested *C*. *sativa* (380 ± 77) *vs*. non-infested *V*. *faba* (544 ± 93) (Wilcoxon rank sum test, *W =* 72, *P* = 0.06); *A*. *fabae*-infested *C*. *sativa* (286 ± 44) *vs*. non-infested *V*. *faba* (504 ± 152) *(*Wilcoxon rank sum test, *W =* 44, *P* = 0.15); non-infested *C*. *sativa* (263 ± 102) *vs*. *A*. *fabae*-infested *V*. *faba* (397 ± 75) (Wilcoxon rank sum test, *W =* 69, *P* = 0.20); *A*. *fabae*-infested *C*. *sativa* (480 ± 57) *vs*. *A*. *faba*e-infested *V*. *faba* (661 ± 88) (Wilcoxon rank sum test, *W =* 257, *P* = 0.08).

### Bioassay 2: Host recognition and acceptance

The first choice was significantly in favour of *A*. *fabae* on *C*. *sativa* (Table 1). The number of Antennal Examination, Abdomen Bending and Ovipositor Insertion was significantly greater on *A*. *fabae* on *C*. *sativa* (Table 1, S5 Table). Latency time was not significantly different between *A*. *fabae* reared on *C*. *sativa* and aphids reared on *V*. *faba* (Table 1). Out of the 21 females that first chose the *A*. *fabae* on *C*. *sativa*, 10 left this patch and moved to *A*. *fabae* on *V*. *faba* (47.62%). Conversely, out of the nine females that first chose *A*. *fabae* on *V*. *faba*, three left this patch and moved to *A*. *fabae* on *C*. *sativa* (33.33%). No significant difference was found between these two percentages (Fisher’s exact test, *P =* 0.73). When considering all the behavioural items on *C*. *sativa* (n = 214), only 4.67% of these realised items were followed by a shift on *V*. *faba* (S6 Table). Conversely 6.60% of the items on *V*. *faba* (n = 91) were followed by a shift on *C*. *sativa*. These percentages were not significantly different (χ^2^ = 0.33, *P* = 0.57).

**Table 1:** Host recognition and acceptance behaviour of *Aphidius matricariae* females (n = 30) on *Aphis fabae* reared either on *C*. *sativa* or *V*. *faba*.

Behavioural parameters	*C*.* sativa-A*.* fabae*	*V*.* faba-A*.* fabae*	Statistics	df	*P*
**Latency time (s; mean ± SEM)**	92.80 ± 19.72	146.90 ± 28.45	*U* = 70.5		0.29
**1st choice**	21	9	*χ* ^2^ = 4.033		0.045
**AE (mean ± SEM)**	5.33 ± 0.58	2.95 ± 0.61	*t* = -2.546	38.815	0.015
**AB (mean ± SEM)**	1.58 ± 0.33	0.2 ± 0.06	*t* = -3.943	26.716	< 0.001
**OI (mean ± SEM)**	2.00 ± 0.43	1.35 ± 0.34	*t* = -2.107	35.58	0.042
**%_OV**	41.4%	69.8%	*χ* ^2^ = 1.165		0.28

(AE: number of Antennal Examination; AB: number of Abdomen Bending; OI: number of Ovipositor Insertion;%_OV: percentage of true Oviposition; *U*: Mann-Whitney rank-sum tests; *χ* ^2^: Chi-square test and *t*: student *t-*tests).

**Fig 3:**
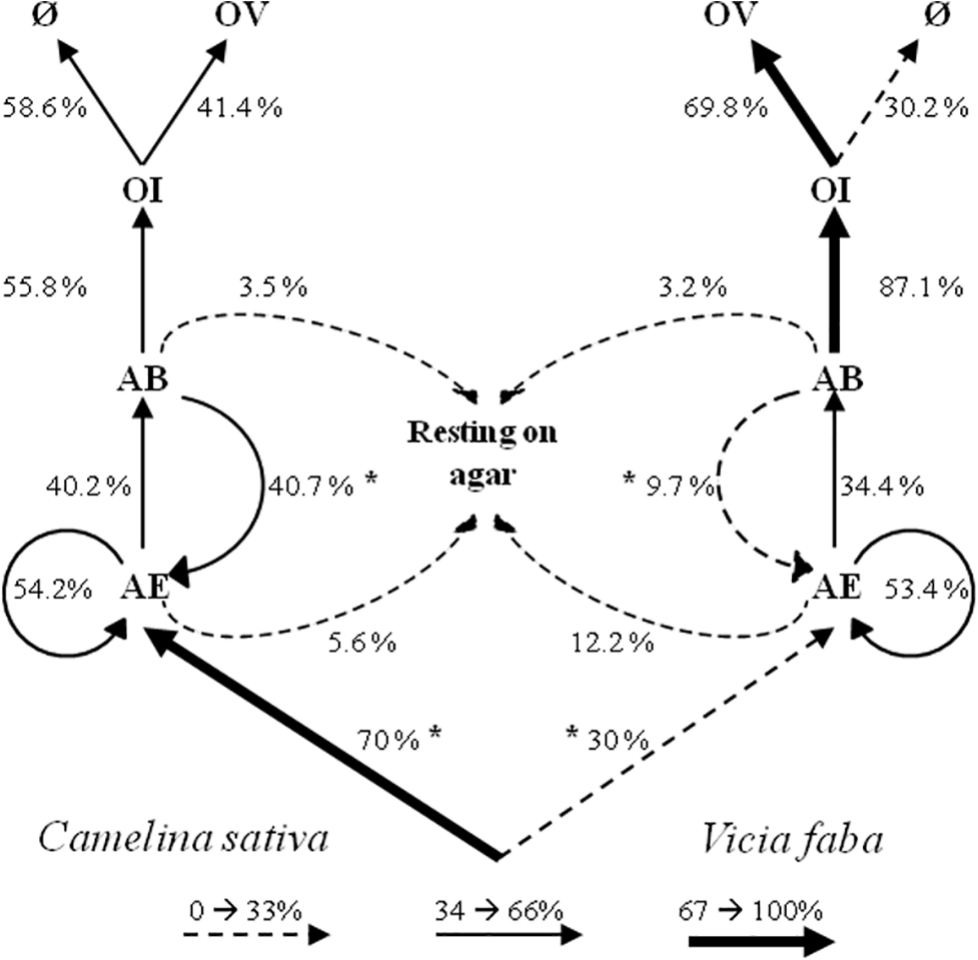
Ethogram of *Aphidius matricariae* attack behaviour facing *Aphis fabae* on two different host plants, *Camelina sativa* and *Vicia faba*, presented in a dual-choice attack rate assay. The width of each line is proportional to the transitional probability of occurrence between two behavioural items. Asterisks indicate significant differences (* *P* < 0.05) when comparing the probabilities of occurrence of one item between the two plant-aphid complexes (Fisher’s exact tests). (AE: Antennal Examination, AB: Abdomen Bending, OI: Ovipositor Insertion, OV: Oviposition and Ø: No egg found).

The probability that Abdomen Bending (AB) was followed by Antennal Examination (AE) was four times greater on the *C*. *sativa-A*. *fabae* complex (Fisher’s exact test, *P* = 0.022), whereas on the *V*. *faba-A*. *fabae* complex, it was more likely to be followed by Ovipositor Insertion (OI) (Fig 3).

### Bioassay 3: Host suitability

Three-day old *A*. *fabae* nymphs had a significantly smaller size when reared on *C*. *sativa* compared to *V*. *faba* (mm; mean ± SEM) (0.80 ± 0.02 and 0.94 ± 0.02, respectively, Student *t*-test, *t* = -5.73; *P* < 0.001; S7 Table). Pre-nymphal and total developmental times of *A*. *matricariae* parasitoids were significantly longer (*ca*. one day) on *C*. *sativa* than on *V*. *faba*, whereas no difference was found for nymphal developmental time (Table 2). On *C*. *sativa*, parasitoid mummification and emergence rates were significantly lower (*ca*. 50%) than on *V*. *faba* (Table 2). Mummy length and parasitoid size (hind tibia length) were significantly smaller on *C*. *sativa* than on *V*. *faba*. No significant difference was found between *C*. *sativa* and *V*. *faba* for *A*. *matricariae* females egg load.

**Table 2:** Effect of the plant-*Aphis fabae* complex on several life history traits of the parasitoid *Aphidius matricariae*.

	*Camelina sativa*				*Vicia faba*
Life history traits parameters	n = 21	Statistics	df	*P*	n = 19
**Pre-nymphal development (d; mean ± SEM)**	8.59 ± 0.18	*t* = 43.719	18	< 0.001	7.63 ± 0.17
**Nymphal development (d; mean ± SEM)**	5.73 ± 0.15	*t* = -0.232	37.10	0.82	5.68 ± 0.11
**Total development (d; mean ± SEM)**	14.32 ± 0.26	*t* = -2.899	38.95	0.006	13.32 ± 0.23
**Mummy length (mm; mean ± SEM)**	1.34 ± 0.04	*t* = 3.504	38.26	0.001	1.57 ± 0.05
**Hind tibia length (mm; mean ± SEM)**	0.42 ± 0.01	*t* = 4.568	33.50	< 0.001	0.49 ± 0.01
**Egg load (mean ± SEM)**	115.32 ± 8.78	*t* = 0.896	30.10	0.38	124.16 ± 4.50
**Mummification rate (%)**	19.33%	*F =* 0.018		0.018	39.60%
**Emergence rate (%)**	36.67%	*F =* 0.006		0.006	73.90%

(*t*: student *t*-tests and *F*: Fisher’s exact tests).

## Discussion

We showed that *A*. *matricariae* females exhibited an initial preference for the plant-aphid complex that would not allow the best progeny performance, consequently invalidating the "Mother knows best" hypothesis. Indeed, in our study, *A*. *matricariae* females preferred to orientate towards the *C*. *sativa-A*. *fabae* complex and showed a greater interest (Antennation Examination and Abdomen Bending) for aphids on camelina whereas aphids were more readily accepted and suitable for parasitoid development when reared on *V*. *faba*. Such paradoxical choice, opposing optimal foraging and optimal oviposition, can easily be explained in phytophagous insects where females may choose to feed and oviposit on hosts that enhance their own adult performance (realised fecundity) but not their offspring performance (survival and development time) [38]. This was empirically validated in phytophagous grass miner females [39]. It has also been reported in the generalist parasitoid *Aphidius ervi* whose females were preferentially attracted by third and fourth instars hosts while their offspring performance was maximized on second instars hosts [5]. However, these studies testing the optimal oviposition theory have explored a direct measurement of parasitoid preference that involved only two trophic levels (*i*.*e*. herbivore and parasitoid) and therefore excluded the first trophic level (*i*.*e*. the plant). Few studies have included the first trophic level in their evaluation of the impact of the host plant quality on the preference and performance of parasitoid wasps. Indeed, [40] and [41] reported that the specialist parasitoid *Cotesia glomerata* preferred to alight on *Brassica nigra*-*Pieris brassicae* complexes that were not co-infested with cabbage root flies, and that this behaviour was correlated with offspring performance [42]. In addition, plant preferences by specialist parasitoids *Diadegma semiclausum* and *C*. *glomerata* for their respective lepidopteran hosts, *Plutella xylostella* and *P*. *brassicae*, were positively correlated with plant quality for offspring performance, which led the authors to state that “Mother knows best” [43]. Indeed, they showed that parasitoid wasps could innately predict host quality on the basis of plant odours. Conversely to these studies on tri-trophic systems, our work is not in accordance with the "Mother knows best" hypothesis. Explanations for the mismatch between mother preference and host suitability can include threat of hyperparasitoids, host defence, and learning of plant-host complex cues [5]. In our study we used naïve parasitoid females, with no oviposition nor olfactive experience. Parasitoids are known to exhibit associative learning of volatile compounds emitted by the plant during oviposition, subsequently allowing them to select more accurately a suitable host-plant complex according to plant odour [44]. The benefits of learning ability are correlated with the variability of host resources (polyphagous) and the lifetime of the female [45]. Within the framework of our study model and given the results of the finals phases of host selection, it is expected that the disruption observed in the host selection process would decrease with the age/experience of female parasitoid.

Parasitoid reproductive success is closely correlated with the female’s ability to find hosts [8]; therefore, parasitoids have evolved efficient foraging strategies to locate hosts in complex environments. Many aphid parasitoids respond weakly to plant or aphid odours alone [46,47], but use synomones to locate aphid-infested plants [48,49]. Although *A*. *matricariae* did not discriminate between its two host plants, the preference for one of the two plant-host complexes could be explained by differences in volatile compound blends emitted. This generally allows parasitoids to discriminate between species of plants [47,50] and/or species of herbivores [10,32]. A meta-analysis [51] showed a strong correlation between preference and performance in oligophagous species of herbivores, but not in polyphagous species. Our results and the literature previously cited suggest that this correlation could be transferred to the third trophic level (*i*.*e*. between generalist and specialist parasitoids). The ability of parasitoids to exploit plant-derived volatiles is higher when host ranges are narrow (*i*.*e*. specialist parasitoids) [14].

A number of the compounds released are common to most plants and are referred as green leaf volatiles (GLV). However, the composition of the entire blend and the concentrations of specific compounds differ based on plant and herbivore species. Those chemicals that promote the effectiveness of natural enemies involve volatile compounds produced in response to herbivore feeding damage, so-called herbivore induced-plant volatiles (HIPV), and are known to be attractive to parasitoids and predators of arthropod herbivores [10].

In our study, *A*. *matricariae* females seemed to be sensitive to HIPV because they showed a preference for the plant-aphid complex in comparison to a non-infested plant. Similar responses by *A*. *matricariae* to plant-aphid complex were demonstrated [52] and for *A*. *ervi* [32]. The quantity of emitted and perceived plant volatiles is important for parasitoid females when searching for herbivorous hosts [53]. Therefore, the preference for the *C*. *sativa-A*. *fabae* complex could possibly be explained by supposing that it had a different GLV emission profile than the *V*. *faba-A*. *fabae* one. Indeed, GLVs were found to have positive effects on host location by parasitoids [49,54] and to be important for mediating parasitoid attraction to herbivore-damaged Brassicaceae [55].

Once host location is achieved, upon host encounter by the parasitoid female, effective detection of the host occurs during ‘antennal palpation’ [34] and is based on physical and chemical cues acting at short range or by contact [48]. Some behavioural items linked to host recognition (AE and AB) were enhanced on *A*. *fabae* on *C*. *sativa*, but the numbers of OI, allowing the wasp to assess host quality before oviposition, were identical on aphids reared on both plants. Nevertheless, the ethogram of *A*. *matricariae* attack behaviour on the two plant-aphid patches emphasizes that the host recognition step was more effective on *A*. *fabae* on *V*. *faba*, with increased transitions between AB and OI, and consequently fewer returns from AB to AE, compared to *A*. *fabae* on *C*. *sativa*. These results suggest an alteration in the host selection process on the *C*. *sativa-A*. *fabae* complex, which is confirmed by the lower oviposition rate measured on this complex. Host acceptance for *A*. *matricariae* females seems to be a function of stimuli firstly perceived during AE, resulting in some rejection before OI, and finally during OI, when host quality is assessed before oviposition. Although *V*. *faba* leaf area was slightly greater, it is unlikely that this difference could have had a decisive influence on the behaviour of the parasitoids. Indeed, at this spatial scale and at this stage of the host selection process, parasitoid females predominantly use cues emanating from the hosts themselves even if plants may also play a role in the host selection behaviour. Changes in host acceptance that depend on the host plant have already been recorded in other aphid parasitoids. For example in laboratory experiment, *L*. *testaceipes* oviposited into more aphids on mungbean than on cotton [56]. Overall in our study, the two plants were suitable with oviposition occurring on both species, but host acceptance of *A*. *matricariae* was enhanced on *V*. *faba* compared to *C*. *sativa*.

In order to see if such a final decision to preferentially oviposit in *A*. *fabae* on *V*. *faba* was linked to a better performance of the progeny on this complex, a controlled oviposition bioassay was performed. Our results indicate that parasitoid fitness was higher when they had developed on *V*. *faba* compared to *C*. *sativa*. This is in accordance with studies in other systems where the size of the emerging solitary parasitoid, used as a fitness proxy, is correlated to “host quality” (size, age, stage and diet) (for review, see [8,57]). Indeed, in our study, aphid hosts developing on *V*. *faba* were bigger than those developing on *C*. *sativa* and therefore offered better quality for parasitoid development. This size difference observed between aphids feeding on *C*. *sativa* and *V*. *faba* could be due to different amino acid composition [58]. Indeed, the growth and fecundity of phytophagous insects are generally limited by nitrogen, in terms of quantity and quality (*i*.*e*. composition). The latter occurs because aphids lack the ability to synthesize nine 'essential' amino acids; and if the concentration of one of those is in short supply, protein synthesis and animal growth are constrained [59]. Various studies with other koinobiont parasitoids have reported that parasitoid size (which is often correlated with parasitoid fecundity) is an increasing function of host size or stage at oviposition [15], with bigger hosts usually representing a greater resource [60]. However, this link was not found in our study, in which no significant difference of parasitoid egg load was observed.

The lower performance of *A*. *matricariae* on the *C*. *sativa*-*A*. *fabae* complex could also be partly explained by the presence of camelina-specific secondary compounds that may be harmful to the developing parasitoid larvae. Few studies have investigated the effects of secondary plant chemistry mediated through the host on parasitoid performance [15,16]. Camelina tissues exhibit different glucosinolates [61], Brassicaceae secondary metabolites that may negatively affect the fitness of parasitoids [16,55]. Moreover, the presence of Camalexin was identified in camelina [62], a secondary compound reducing the performance of *Myzus persicae*, a generalist aphid species [63]. Performance of the host and that of its parasitoid are often positively correlated [64] but the adverse effects of food plant characteristics on insect performance are usually less pronounced in the parasitoid than in its herbivore host. Here, camelina plants seemed to have more drastic effects on *A*. *matricariae* than on *A*. *fabae*: the overall fitness of parasitoids was reduced whereas intrinsic rate of natural increase (*r*_*m*_) was equivalent [24] and only sizes were affected in aphid hosts. Ultimately, the sharp decline in parasitoids fitness could also be due to the host plant range of *A*. *fabae* hosts. Indeed, parasitoids attacking generalist hosts have been shown to be more strongly affected by the herbivore’s diet than parasitoids that attack specialist hosts [64].

## Conclusion

These findings may have important implications for agricultural production in sustainable systems. The presence of camelina induced a disruption of the initial foraging decisions of *A*. *matricariae* females towards the *V*. *faba*-*A*. *fabae* complex, potentially impairing the top-down regulation of the black bean aphid in such an intercropping association. From the aphid perspective, camelina seemed to be an 'enemy free-space', as described by [65] stating that plants can provide an ecological refuge for herbivores by allowing them to chemically or physically escape their natural enemies. Based on the first phases of host selection by parasitoids, the *C*. *sativa*-*A*. *fabae* complex may ultimately be considered as an ecological trap for *A*. *matricariae* (*i*.*e*. a low-quality habitat that organisms prefer over superior habitats) [66]. This concept has been set for animals that make errors in habitat assessment as a result of some mismatch between the environmental cues they use to select habitats and actual habitat quality (for review, see [67]).

By demonstrating that *A*. *matricariae* females are able to discriminate the aphid host that offers the highest fitness to their offspring but select beforehand the least suitable plant-aphid complex, this study provides key insight into the disruption in their host selection behaviour potentially triggered by diverse habitats.

## Supporting Information

S1 Table Bioassay 1: Habitat and host-plant location—Non-infested *C*. *sativa* vs. non-infested *V*. *faba*. Responses made by *Aphidius matricariae* females when presented with a choice between non-infested *C*. *sativa* vs. non-infested *V*. *faba*. Females that landed on either plant within 20 min were considered as “responding” females (Response = 1) whereas they were considered as “non-responding” when they left the take-off plateform but did not choose any target (Response = 0). If they did not leave the take-off plateform within 20 min they were discarded (Response = D). Times from introduction to first choice by responding females were recorded (latency time).
(DOCX)

S2 Table Bioassay 1: Habitat and host-plant location—*A*. *fabae*-infested *C*. *sativa* vs. non-infested *V*. *faba*. Responses made by *Aphidius matricariae* females when presented with a choice between *A*. *fabae*-infested *C*. *sativa* vs. non-infested *V*. *faba*. Females that landed on either plant within 20 min were considered as “responding” females (Response = 1) whereas they were considered as “non-responding” when they left the take-off plateform but did not choose any target (Response = 0). If they did not leave the take-off plateform within 20 min they were discarded (Response = D). Times from introduction to first choice by responding females were recorded (latency time).
(DOCX)

S3 Table Bioassay 1: Habitat and host-plant location—Non-infested *C*. *sativa* vs. *A*. *fabae*-infested *V*. *faba*. Responses made by *Aphidius matricariae* females when presented with a choice between non-infested *C*. *sativa* vs. *A*. *fabae*-infested *V*. *faba*. Females that landed on either plant within 20 min were considered as “responding” females (Response = 1) whereas they were considered as “non-responding” when they left the take-off plateform but did not choose any target (Response = 0). If they did not leave the take-off plateform within 20 min they were discarded (Response = D). Times from introduction to first choice by responding females were recorded (latency time).
(DOCX)

S4 Table Bioassay 1: Habitat and host-plant location—*A*. *fabae*-infested *C*. *sativa* vs. *A*. *fabae*-infested *V*. *faba*. Responses made by *Aphidius matricariae* females when presented with a choice between *A*. *fabae*-infested *C*. *sativa* vs. *A*. *fabae*-infested *V*. *faba*. Females that landed on either plant within 20 min were considered as “responding” females (Response = 1) whereas they were considered as “non-responding” when they left the take-off plateform but did not choose any target (Response = 0). If they did not leave the take-off plateform within 20 min they were discarded (Response = D). Times from introduction to first choice by responding females were recorded (latency time).
(DOCX)

S5 Table Bioassay 2: Host recognition and acceptance behaviour of *Aphidius matricariae* females on *Aphis fabae* reared on either *C*. *sativa* or *V*. *faba*. *A*. *matricariae* females were individually tested in an attack rate bioassay where they were presented with a choice between 10 *A*. *fabae* reared on *V*. *faba* deposited on *V*. *faba* leaves and 10 *A*. *fabae* reared on *C*. *sativa* deposited on *C*. *sativa* leaves. Observation of the female wasps lasted for 10 minutes after their introduction. Different behavioural items were recorded and their frequencies are reported in the table below (AE: number of Antennal Examination, AB: number of Abdomen Bending, OI: number of Ovipositor Insertion). The time before the first recorded behavioural item (latency time) and the first choice (aphid patch which was reached first by the parasitoid) are also presented in the table. Immediately after the attack rate bioassay, all stung aphids were dissected and the number of parasitoid eggs (Eggs) recorded.
(DOCX)

S6 Table Numbers and percentages of behavioural items (AE, AB, OI or All) that were followed by a shift from *C*. *sativa* to *V*. *faba* or from *V*. *faba* to *C*. *sativa*. (AE: number of Antennal Examination, AB: number of Abdomen Bending, OI: number of Ovipositor Insertion). The total numbers of behavioural items performed are indicated in brackets.
(DOCX)

S7 Table Bioassay 3: Host suitability: Effect of the plant- *Aphis fabae* complex on several life history traits of the parasitoid *Aphidius matricariae*. Parasitoids’ life-history traits were measured on the females wasps that had developed on *A*. *fabae* aphids that were reared either on *Camelina sativa* or on *Vicia faba*. For each emerging female individual the following parameters were measured: Tibia length (in cm) and Egg load (No. eggs); Mummy size (length in cm); Pre-nymphal developmental time (from oviposition to mummification) in days; Nymphal developmental time (from mummification to adult emergence) in days; Total developmental time (from oviposition to adult emergence) in days.
(DOCX)
