# Supplementary material for: Aphid Parasitoid Mothers Don't Always Know Best through the Whole Host Selection Process
Source: PLoS One. 2015 Aug 13;10(8):e0135661. doi: 10.1371/journal.pone.0135661 (PMC4535949; doi:10.1371/journal.pone.0135661)
Supplement: S1 Table — Responses made by Aphidius matricariae females when presented with a choice between non-infested C. sativa vs. non-infested V. faba. Females that landed on either plant within 20 min were considered as “responding” females (Response = 1) whereas they were considered as “non-responding” when they left the take-off plateform but did not choose any target (Response = 0). If they did not leave the take-off plateform within 20 min they were discarded (Response = D). Times from introduction to first choice by responding females were recorded (latency time). (DOCX) [file pone.0135661.s001.docx]

**S1 Table. Bioassay 1: Habitat and host-plant location - Non-infested *C. sativa* vs. non-infested *V. faba***Responses made by *Aphidius matricariae* females when presented with a choice between non-infested *C. sativa* vs. non-infested *V. faba.* Females that landed on either plant within 20 min were considered as “responding” females (Response = 1) whereas they were considered as “non-responding” when they left the take-off plateform but did not choose any target (Response = 0). If they did not leave the take-off plateform within 20 min they were discarded (Response = D). Times from introduction to first choice by responding females were recorded (latency time).

| **Individual** | **Response** | **Choice** | **Latency time (s)** |
| --- | --- | --- | --- |
| 1 | 0 | ∅ | - |
| 2 | 1 | *Vicia faba* | 162 |
| 3 | 1 | *Vicia faba* | 461 |
| 4 | 1 | *Vicia faba* | 1140 |
| 5 | 1 | *Camelina sativa* | 109 |
| 6 | 1 | *Camelina sativa* | 92 |
| 7 | 1 | *Camelina sativa* | 974 |
| 8 | 1 | *Camelina sativa* | 160 |
| 9 | 0 | ∅ | - |
| 10 | 1 | *Camelina sativa* | 243 |
| 11 | 1 | *Vicia faba* | 288 |
| 12 | 1 | *Camelina sativa* | 138 |
| 13 | 1 | *Camelina sativa* | 185 |
| 14 | 1 | *Vicia faba* | 312 |
| 15 | 1 | *Vicia faba* | 263 |
| 16 | 0 | ∅ | - |
| 17 | 1 | *Camelina sativa* | 568 |
| 18 | 0 | ∅ | - |
| 19 | 1 | *Vicia faba* | 442 |
| 20 | 0 | ∅ | - |
| 21 | 0 | ∅ | - |
| 22 | 1 | *Vicia faba* | 338 |
| 23 | 1 | *Vicia faba* | 418 |
| 24 | D |  | - |
| 25 | 0 | ∅ | - |
| 26 | 1 | *Camelina sativa* | 146 |
| 27 | 1 | *Vicia faba* | 949 |
| 28 | 0 | ∅ | - |
| 29 | 0 | ∅ | - |
| 30 | 1 | *Vicia faba* | NA |
| 31 | 0 | ∅ | - |
| 32 | D | - | - |
| 33 | 1 | *Camelina sativa* | 278 |
| 34 | 0 | ∅ | - |
| 35 | 1 | *Camelina sativa* | 347 |
| 36 | 0 | ∅ | - |
| 37 | 0 | ∅ | - |
| 38 | 1 | *Camelina sativa* | 1020 |
| 39 | 1 | *Vicia faba* | 1120 |
| 40 | 1 | *Vicia faba* | 315 |
| 41 | D | - | - |
| 42 | 0 | ∅ | - |
| 43 | 1 | *Camelina sativa* | 463 |
| 44 | 0 | ∅ | - |
| 45 | D | - | - |
| 46 | 0 | ∅ | - |
| 47 | 1 | *Camelina sativa* | 209 |
| 48 | 1 | *Camelina sativa* | 141 |
| 49 | 1 | *Vicia faba* | 377 |
| 50 | 1 | *Camelina sativa* | 452 |
| 51 | 0 | ∅ | - |
| 52 | 1 | *Vicia faba* | 1029 |
| 53 | 1 | *Camelina sativa* | 942 |
