# Supplementary material for: Aphid Parasitoid Mothers Don't Always Know Best through the Whole Host Selection Process
Source: PLoS One. 2015 Aug 13;10(8):e0135661. doi: 10.1371/journal.pone.0135661 (PMC4535949; doi:10.1371/journal.pone.0135661)
Supplement: S2 Table — Responses made by Aphidius matricariae females when presented with a choice between A. fabae-infested C. sativa vs. non-infested V. faba. Females that landed on either plant within 20 min were considered as “responding” females (Response = 1) whereas they were considered as “non-responding” when they left the take-off plateform but did not choose any target (Response = 0). If they did not leave the take-off plateform within 20 min they were discarded (Response = D). Times from introduction to first choice by responding females were recorded (latency time). (DOCX) [file pone.0135661.s002.docx]

**S2 Table. Bioassay 1: Habitat and host-plant location - *A. fabae*-infested *C. sativa* vs. non-infested *V. faba***Responses made by *Aphidius matricariae* females when presented with a choice between *A*. *fabae*-infested *C. sativa* vs. non-infested *V. faba.* Females that landed on either plant within 20 min were considered as “responding” females (Response = 1) whereas they were considered as “non-responding” when they left the take-off plateform but did not choose any target (Response = 0). If they did not leave the take-off plateform within 20 min they were discarded (Response = D). Times from introduction to first choice by responding females were recorded (latency time).

| **Individual** | **Response** | **Choice** | **Latency time (s)** |
| --- | --- | --- | --- |
| 1 | 1 | *Vicia faba* | 146 |
| 2 | 1 | *Camelina sativa* | 318 |
| 3 | 1 | *Camelina sativa* | 37 |
| 4 | 1 | *Camelina sativa* | 398 |
| 5 | 0 | ∅ | - |
| 6 | 1 | *Camelina sativa* | 172 |
| 7 | 1 | *Vicia faba* | 537 |
| 8 | 1 | *Camelina sativa* | 635 |
| 9 | 0 | ∅ | - |
| 10 | 0 | ∅ | - |
| 11 | 1 | *Camelina sativa* | 587 |
| 12 | 1 | *Camelina sativa* | 211 |
| 13 | 1 | *Vicia faba* | 320 |
| 14 | 1 | *Camelina sativa* | 300 |
| 15 | 1 | *Camelina sativa* | 37 |
| 16 | 1 | *Vicia faba* | 1170 |
| 17 | 1 | *Camelina sativa* | 142 |
| 18 | D | - | - |
| 19 | 1 | *Camelina sativa* | 348 |
| 20 | D | - | - |
| 21 | 1 | *Camelina sativa* | 271 |
| 22 | 1 | *Camelina sativa* | 79 |
| 23 | 1 | *Camelina sativa* | 628 |
| 24 | D | - | - |
| 25 | 0 | ∅ | - |
| 26 | 1 | *Camelina sativa* | 32 |
| 27 | 1 | *Camelina sativa* | 213 |
| 28 | 1 | *Camelina sativa* | 72 |
| 29 | 0 | ∅ | - |
| 30 | D | - | - |
| 31 | 1 | *Camelina sativa* | 268 |
| 32 | 1 | *Camelina sativa* | 339 |
| 33 | 1 | *Camelina sativa* | 241 |
| 34 | 0 | ∅ | - |
| 35 | 1 | *Vicia faba* | 615 |
| 36 | 1 | *Camelina sativa* | 77 |
| 37 | 1 | *Camelina sativa* | 800 |
| 38 | D | - | - |
| 39 | D | - | - |
| 40 | 1 | *Vicia faba* | 237 |
| 41 | 1 | *Camelina sativa* | 137 |
| 42 | 1 | *Camelina sativa* | 517 |
| 43 | 0 | ∅ | - |
| 44 | D | - | - |
| 45 | 0 | ∅ | - |
