# Supplementary material for: Aphid Parasitoid Mothers Don't Always Know Best through the Whole Host Selection Process
Source: PLoS One. 2015 Aug 13;10(8):e0135661. doi: 10.1371/journal.pone.0135661 (PMC4535949; doi:10.1371/journal.pone.0135661)
Supplement: S3 Table — Responses made by Aphidius matricariae females when presented with a choice between non-infested C. sativa vs. A. fabae-infested V. faba. Females that landed on either plant within 20 min were considered as “responding” females (Response = 1) whereas they were considered as “non-responding” when they left the take-off plateform but did not choose any target (Response = 0). If they did not leave the take-off plateform within 20 min they were discarded (Response = D). Times from introduction to first choice by responding females were recorded (latency time). (DOCX) [file pone.0135661.s003.docx]

**S3 Table. Bioassay 1: Habitat and host-plant location - Non-infested *C. sativa* vs. *A. fabae*-infested *V. faba***

Responses made by *Aphidius matricariae* females when presented with a choice between non-infested *C. sativa* vs. *A. fabae*-infested *V. faba.* Females that landed on either plant within 20 min were considered as “responding” females (Response = 1) whereas they were considered as “non-responding” when they left the take-off plateform but did not choose any target (Response = 0). If they did not leave the take-off plateform within 20 min they were discarded (Response = D). Times from introduction to first choice by responding females were recorded (latency time).

| **Individual** | **Response** | **Choice** | **Latency time (s)** |
| --- | --- | --- | --- |
| 1 | 0 | ∅ | - |
| 2 | 0 | ∅ | - |
| 3 | 1 | *Vicia faba* | 105 |
| 4 | D | - | - |
| 5 | 0 | ∅ | - |
| 6 | 1 | *Vicia faba* | 36 |
| 7 | 1 | *Vicia faba* | 212 |
| 8 | 0 | ∅ | - |
| 9 | 1 | *Camelina sativa* | 247 |
| 10 | 1 | *Vicia faba* | 154 |
| 11 | 1 | *Camelina sativa* | 17 |
| 12 | D | - | - |
| 13 | D | - | - |
| 14 | 0 | ∅ | - |
| 15 | 1 | *Vicia faba* | 465 |
| 16 | 1 | *Vicia faba* | 66 |
| 17 | 1 | *Vicia faba* | 268 |
| 18 | D | - | - |
| 19 | 1 | *Vicia faba* | 219 |
| 20 | 1 | *Vicia faba* | 385 |
| 21 | 1 | *Vicia faba* | 980 |
| 22 | 1 | *Camelina sativa* | 870 |
| 23 | 1 | *Vicia faba* | 361 |
| 24 | 1 | *Vicia faba* | 907 |
| 25 | 1 | *Vicia faba* | 110 |
| 26 | 1 | *Vicia faba* | 376 |
| 27 | 1 | *Vicia faba* | 351 |
| 28 | 1 | *Vicia faba* | 818 |
| 29 | 1 | *Camelina sativa* | 45 |
| 30 | 0 | ∅ | - |
| 31 | 1 | *Vicia faba* | 1072 |
| 32 | D | - | - |
| 33 | 0 | ∅ | - |
| 34 | 1 | *Camelina sativa* | 20 |
| 35 | 1 | *Camelina sativa* | 517 |
| 36 | 1 | *Vicia faba* | 150 |
| 37 | D | - | - |
| 38 | 1 | *Camelina sativa* | 116 |
| 39 | 1 | *Vicia faba* | 92 |
| 40 | D | - | - |
| 41 | D | - | - |
| 42 | 1 | *Camelina sativa* | 528 |
| 43 | 1 | *Camelina sativa* | 9 |
| 44 | 0 | ∅ | - |
| 45 | 1 | *Vicia faba* | 1125 |
| 46 | 0 | ∅ | - |
| 47 | 1 | *Vicia faba* | 77 |
| 48 | 1 | *Vicia faba* | 408 |
