# Supplementary material for: Aphid Parasitoid Mothers Don't Always Know Best through the Whole Host Selection Process
Source: PLoS One. 2015 Aug 13;10(8):e0135661. doi: 10.1371/journal.pone.0135661 (PMC4535949; doi:10.1371/journal.pone.0135661)
Supplement: S4 Table — Responses made by Aphidius matricariae females when presented with a choice between A. fabae-infested C. sativa vs. A. fabae-infested V. faba. Females that landed on either plant within 20 min were considered as “responding” females (Response = 1) whereas they were considered as “non-responding” when they left the take-off plateform but did not choose any target (Response = 0). If they did not leave the take-off plateform within 20 min they were discarded (Response = D). Times from introduction to first choice by responding females were recorded (latency time). (DOCX) [file pone.0135661.s004.docx]

**S4 Table. Bioassay 1: Habitat and host-plant location - *A. fabae*-infested *C. sativa* vs. *A. fabae*-infested *V. faba***

Responses made by *Aphidius matricariae* females when presented with a choice between *A. fabae*-infested *C. sativa* vs. *A. fabae*-infested *V. faba.* Females that landed on either plant within 20 min were considered as “responding” females (Response = 1) whereas they were considered as “non-responding” when they left the take-off plateform but did not choose any target (Response = 0). If they did not leave the take-off plateform within 20 min they were discarded (Response = D). Times from introduction to first choice by responding females were recorded (latency time).

| **Individual** | **Response** | **Choice** | **Latency time (s)** |
| --- | --- | --- | --- |
| 1 | D | - | - |
| 2 | D | - | - |
| 3 | 0 | ∅ | - |
| 4 | 1 | *Vicia faba* | 420 |
| 5 | 1 | *Camelina sativa* | 60 |
| 6 | 1 | *Vicia faba* | 240 |
| 7 | 1 | *Camelina sativa* | 150 |
| 8 | 1 | *Camelina sativa* | 97 |
| 9 | 0 | ∅ | - |
| 10 | D | - | - |
| 11 | 0 | ∅ | - |
| 12 | 0 | ∅ | - |
| 13 | 1 | *Camelina sativa* | 452 |
| 14 | 1 | *Camelina sativa* | 860 |
| 15 | 1 | *Camelina sativa* | 275 |
| 16 | 1 | *Camelina sativa* | 132 |
| 17 | D | - | - |
| 18 | 0 | ∅ | - |
| 19 | 1 | *Camelina sativa* | 37 |
| 20 | 1 | *Camelina sativa* | 862 |
| 21 | 0 | ∅ | - |
| 22 | 1 | *Camelina sativa* | 117 |
| 23 | 0 | ∅ | - |
| 24 | 1 | *Vicia faba* | 630 |
| 25 | 1 | *Camelina sativa* | 450 |
| 26 | 1 | *Camelina sativa* | 470 |
| 27 | 0 | ∅ | - |
| 28 | 1 | *Camelina sativa* | 1170 |
| 29 | 0 | ∅ | - |
| 30 | 1 | *Camelina sativa* | 65 |
| 31 | 1 | *Camelina sativa* | 641 |
| 32 | 0 | ∅ | - |
| 33 | 0 | ∅ | - |
| 34 | 1 | *Camelina sativa* | 103 |
| 35 | 1 | *Vicia faba* | 958 |
| 36 | 0 | ∅ | - |
| 37 | 0 | ∅ | - |
| 38 | 1 | *Vicia faba* | 349 |
| 39 | 1 | *Vicia faba* | 1152 |
| 40 | 1 | *Vicia faba* | 162 |
| 41 | 0 | ∅ | - |
| 42 | 1 | *Vicia faba* | 138 |
| 43 | 1 | *Camelina sativa* | 593 |
| 44 | 1 | *Camelina sativa* | 39 |
| 45 | 1 | *Vicia faba* | 1087 |
| 46 | 0 | ∅ | - |
| 47 | 0 | ∅ | - |
| 48 | 1 | *Vicia faba* | 1032 |
| 49 | 0 | ∅ | - |
| 50 | 1 | *Vicia faba* | 139 |
| 51 | 1 | *Camelina sativa* | 382 |
| 52 | 0 | ∅ | - |
| 53 | 1 | *Camelina sativa* | 507 |
| 54 | 1 | *Vicia faba* | 244 |
| 55 | 1 | *Camelina sativa* | 956 |
| 56 | 0 | ∅ | - |
| 57 | 0 | ∅ | - |
| 58 | 0 | ∅ | - |
| 59 | 0 | ∅ | - |
| 60 | 0 | ∅ | - |
| 61 | 0 | ∅ | - |
| 62 | D | - | - |
| 63 | D | - | - |
| 64 | 1 | *Camelina sativa* | 22 |
| 65 | 1 | *Camelina sativa* | 983 |
| 66 | 1 | *Vicia faba* | 840 |
| 67 | 0 | ∅ | - |
| 68 | 1 | *Camelina sativa* | 180 |
| 69 | 0 | ∅ | - |
| 70 | 1 | *Camelina sativa* | 267 |
| 71 | 1 | *Vicia faba* | 1030 |
| 72 | 0 | ∅ | - |
| 73 | 1 | *Vicia faba* | 525 |
| 74 | 1 | *Vicia faba* | 821 |
| 75 | 1 | *Camelina sativa* | 1157 |
| 76 | 1 | *Vicia faba* | 1063 |
| 77 | 0 | ∅ | - |
| 78 | 1 | *Camelina sativa* | 1080 |
| 79 | 1 | *Camelina sativa* | 443 |
| 80 | 1 | *Camelina sativa* | 633 |
| 81 | 1 | *Camelina sativa* | 260 |
| 82 | 1 | *Camelina sativa* | 508 |
| 83 | 0 | ∅ | - |
| 84 | 1 | *Camelina sativa* | 30 |
| 85 | 0 | ∅ | - |
| 86 | 1 | *Camelina sativa* | 487 |
| 87 | 1 | *Camelina sativa* | 582 |
| 88 | 1 | *Vicia faba* | 528 |
| 89 | 1 | *Camelina sativa* | 822 |
| 90 | 1 | *Camelina sativa* | 757 |
| 91 | 1 | *Vicia faba* | 1200 |
| 92 | 1 | *Camelina sativa* | 727 |
| 93 | 1 | *Camelina sativa* | 876 |
| 94 | 1 | *Vicia faba* | NA |
| 95 | 1 | *Camelina sativa* | NA |
| 96 | 1 | *Vicia faba* | NA |
| 97 | 1 | *Vicia faba* | NA |
