# Supplementary material for: Aphid Parasitoid Mothers Don't Always Know Best through the Whole Host Selection Process
Source: PLoS One. 2015 Aug 13;10(8):e0135661. doi: 10.1371/journal.pone.0135661 (PMC4535949; doi:10.1371/journal.pone.0135661)
Supplement: S5 Table — A. matricariae females were individually tested in an attack rate bioassay where they were presented with a choice between 10 A. fabae reared on V. faba deposited on V. faba leaves and 10 A. fabae reared on C. sativa deposited on C. sativa leaves. Observation of the female wasps lasted for 10 minutes after their introduction. Different behavioural items were recorded and their frequencies are reported in the table below (AE: number of Antennal Examination, AB: number of Abdomen Bending, OI: number of Ovipositor Insertion). The time before the first recorded behavioural item (latency time) and the first choice (aphid patch which was reached first by the parasitoid) are also presented in the table. Immediately after the attack rate bioassay, all stung aphids were dissected and the number of parasitoid eggs (Eggs) recorded. (DOCX) [file pone.0135661.s005.docx]

**S5 Table. Bioassay 2: Host recognition and acceptance behaviour of *Aphidius matricariae* females on *Aphis fabae* reared on either *C. sativa* or *V. faba*.**

*A. matricariae* females were individually tested in an attack rate bioassay where they were presented with a choice between 10 *A. fabae* reared on *V. faba* deposited on *V. faba* leaves and 10 *A. fabae* reared on *C. sativa* deposited on *C. sativa* leaves. Observation of the female wasps lasted for 10 minutes after their introduction. Different behavioural items were recorded and their frequencies are reported in the table below (AE: number of Antennal Examination, AB: number of Abdomen Bending, OI: number of Ovipositor Insertion).

The time before the first recorded behavioural item (latency time) and the first choice (aphid patch which was reached first by the parasitoid) are also presented in the table. Immediately after the attack rate bioassay, all stung aphids were dissected and the number of parasitoid eggs (Eggs) recorded.

|  |  |  | ***A. fabae* on *C. sativa*** | | | | ***A. fabae* on *V. faba*** | | | |
| --- | --- | --- | --- | --- | --- | --- | --- | --- | --- | --- |
| **Individual** | **1st choice** | **Latency time (s)** | **AE** | **AB** | **OI** | **Eggs** | **AE** | **AB** | **OI** | **Eggs** |
| A1 | *C. sativa* | 125 | 3 | 1 | 0 | 0 | - | - | - | - |
| A2 | *C. sativa* | 282 | 5 | 0 | 0 | 0 | - | - | - | - |
| A3 | *V. faba* | 139 | - | - | - | - | 7 | 1 | 0 | 0 |
| A4 | *C. sativa* | 13 | 1 | 0 | 2 | 1 | 5 | 0 | 1 | 1 |
| A5 | *C. sativa* | 210 | 7 | 3 | 3 | 1 | 1 | 0 | 2 | 2 |
| A6 | *V. faba* | 90 | 0 | 1 | 6 | 3 | 4 | 0 | 5 | 3 |
| A7 | *V. faba* | 15 | 3 | 2 | 3 | 1 | 3 | 1 | 3 | 2 |
| A8 | *V. faba* | 112 | 3 | 3 | 3 | 2 | 12 | 0 | 1 | 1 |
| A9 | *C. sativa* | 203 | 5 | 1 | 0 | 0 | - | - | - | - |
| A10 | *C. sativa* | 20 | 4 | 0 | 0 | 0 | 3 | 0 | 0 | 0 |
| A11 | *C. sativa* | 179 | 7 | 0 | 0 | 0 | - | - | - | - |
| A12 | *C. sativa* | 39 | 7 | 0 | 0 | 0 | - | - | - | - |
| A13 | *C. sativa* | 34 | 10 | 2 | 2 | 1 | - | - | - | - |
| A14 | *V. faba* | 270 | - | - | - | - | 5 | 0 | 0 | 0 |
| A15 | *C. sativa* | 205 | 7 | 0 | 0 | 0 | - | - | - | - |
| A16 | *V. faba* | 40 | - | - | - | - | 1 | 0 | 2 | 1 |
| A17 | *C. sativa* | 37 | 6 | 0 | 0 | 0 | 2 | 0 | 0 | 0 |
| A18 | *C. sativa* | 13 | 6 | 1 | 0 | 0 | 0 | 0 | 0 | 0 |
| A19 | *C. sativa* | 27 | 5 | 4 | 7 | 3 | - | - | - | - |
| A20 | *C. sativa* | 14 | 4 | 2 | 4 | 2 | 0 | 0 | 4 | 2 |
| A21 | *C. sativa* | 15 | 10 | 5 | 3 | 1 | 1 | 0 | 1 | 0 |
| A22 | *C. sativa* | 71 | 1 | 0 | 1 | 1 | 2 | 0 | 0 | 0 |
| A23 | *V. faba* | 158 | - | - | - | - | 4 | 1 | 4 | 3 |
| A24 | *C. sativa* | 17 | 6 | 1 | 0 | 0 | - | - | - | - |
| A25 | *V. faba* | 292 | - | - | - | - | 2 | 0 | 0 | 0 |
| A26 | *C. sativa* | 23 | 12 | 1 | 2 | 0 | - | - | - | - |
| A27 | *C. sativa* | 159 | 5 | 2 | 4 | 2 | - | - | - | - |
| A28 | *V. faba* | 150 | - | - | - | - | 4 | 0 | 1 | 1 |
| A29 | *C. sativa* | 181 | 6 | 4 | 5 | 1 | 1 | 0 | 3 | 2 |
| A30 | *C. sativa* | 192 | 5 | 5 | 3 | 0 | 2 | 1 | 0 | 0 |
