# Supplementary material for: Aphid Parasitoid Mothers Don't Always Know Best through the Whole Host Selection Process
Source: PLoS One. 2015 Aug 13;10(8):e0135661. doi: 10.1371/journal.pone.0135661 (PMC4535949; doi:10.1371/journal.pone.0135661)
Supplement: S6 Table — (AE: number of Antennal Examination, AB: number of Abdomen Bending, OI: number of Ovipositor Insertion). The total numbers of behavioural items performed are indicated in brackets. (DOCX) [file pone.0135661.s006.docx]

**S6 Table. Numbers and percentages of behavioural items (AE, AB, OI or All) that were followed by a shift from *C. sativa* to *V. faba* or from *V. faba* to *C. sativa*.** (AE: number of Antennal Examination, AB: number of Abdomen Bending, OI: number of Ovipositor Insertion). The total numbers of behavioural items performed are indicated in brackets.

|  | AE | AB | OI | Total |
| --- | --- | --- | --- | --- |
| No. of shifts from *C. sativa* to *V. faba*  % of shifts from *C. sativa* to *V. faba* | 4 (128)  3.125 | 1 (38)  2.63 | 5 (48)  10.42 | 10 (214)  4.67 |
| No. of shifts from *V. faba* to *C. sativa*  % of shifts from *V. faba* to *C. sativa* | 5 (59)  8.47 | 1 (4)  25 | 0 (27)  0 | 6 (91)  6.60 |
