# Supplementary material for: Aphid Parasitoid Mothers Don't Always Know Best through the Whole Host Selection Process
Source: PLoS One. 2015 Aug 13;10(8):e0135661. doi: 10.1371/journal.pone.0135661 (PMC4535949; doi:10.1371/journal.pone.0135661)
Supplement: S7 Table — Parasitoids’ life-history traits were measured on the females wasps that had developed on A. fabae aphids that were reared either on Camelina sativa or on Vicia faba. For each emerging female individual the following parameters were measured: Tibia length (in cm) and Egg load (No. eggs); Mummy size (length in cm); Pre-nymphal developmental time (from oviposition to mummification) in days; Nymphal developmental time (from mummification to adult emergence) in days; Total developmental time (from oviposition to adult emergence) in days. (DOCX) [file pone.0135661.s007.docx]

**S7 Table. Bioassay 3: Host suitability: Effect of the plant- *Aphis fabae* complex on several life history traits of the parasitoid *Aphidius matricariae.***

Parasitoids’ life-history traits were measured on the females wasps that had developed on *A. fabae* aphids that were reared either on *Camelina sativa* or on *Vicia faba*. For each emerging female individual the following parameters were measured: Tibia length (in cm) and Egg load (No. eggs); Mummy size (length in cm); Pre-nymphal developmental time (from oviposition to mummification) in days ; Nymphal developmental time (from mummification to adult emergence) in days ; Total developmental time (from oviposition to adult emergence) in days.

| **Individual** | **Plant** | **Tibia (cm)** | **No. eggs** | **Mummy length (cm)** | **Prenymphal development time(days)** | **Nymphal development time (days)** | **Total development time (days)** |
| --- | --- | --- | --- | --- | --- | --- | --- |
| 1 | *Vicia faba* | 0.05 | 146 | 0.1585 | 9 | 6 | 15 |
| 2 | *Vicia faba* | 0.0533 | 135 | 0.1689 | 9 | 6 | 15 |
| 3 | *Vicia faba* | 0.0468 | 103 | 0.1776 | 8 | 6 | 14 |
| 4 | *Vicia faba* | 0.0526 | 144 | 0.1708 | 9 | 6 | 15 |
| 5 | *Vicia faba* | 0.0509 | 109 | 0.1598 | 7 | 5 | 12 |
| 6 | *Vicia faba* | 0.0546 | 129 | 0.1765 | 8 | 6 | 14 |
| 7 | *Vicia faba* | 0.0491 | 101 | 0.1701 | 7 | 6 | 13 |
| 8 | *Vicia faba* | 0.054 | 130 | 0.1828 | 7 | 6 | 13 |
| 9 | *Vicia faba* | 0.0402 | 101 | 0.1522 | 7 | 5 | 12 |
| 10 | *Vicia faba* | 0.0341 | 92 | 0.1282 | 7 | 5 | 12 |
| 11 | *Vicia faba* | 0.0547 | 156 | 0.1947 | 7 | 5 | 12 |
| 12 | *Vicia faba* | 0.0499 | 151 | 0.1377 | 7 | 6 | 13 |
| 13 | *Vicia faba* | 0.0528 | 126 | 0.1733 | 7 | 6 | 13 |
| 14 | *Vicia faba* | 0.0464 | 137 | 0.1294 | 7 | 6 | 13 |
| 15 | *Vicia faba* | 0.042 | 111 | 0.1324 | 8 | 5 | 13 |
| 16 | *Vicia faba* | 0.0514 | 116 | 0.1383 | 7 | 6 | 13 |
| 17 | *Vicia faba* | 0.0498 | 120 | 0.1596 | 8 | 5 | 13 |
| 18 | *Vicia faba* | 0.0476 | 147 | 0.1285 | 8 | 6 | 14 |
| 19 | *Vicia faba* | 0.0453 | 99 | 0.1467 | 8 | 6 | 14 |
| 20 | *Camelina sativa* | 0.0451 | 157 | 0.1475 | 9 | 5 | 14 |
| 21 | *Camelina sativa* | 0.0465 | 164 | 0.1595 | 7 | 6 | 13 |
| 22 | *Camelina sativa* | 0.0471 | 152 | 0.1714 | 8 | 6 | 14 |
| 23 | *Camelina sativa* | 0.0378 | 90 | 0.1016 | 8 | 5 | 13 |
| 24 | *Camelina sativa* | 0.0433 | 130 | 0.1472 | 9 | 6 | 15 |
| 25 | *Camelina sativa* | 0.0494 | 183 | 0.1646 | 7 | 5 | 12 |
| 26 | *Camelina sativa* | 0.0417 | 114 | 0.1124 | 10 | 7 | 17 |
| 27 | *Camelina sativa* | 0.0424 | 117 | 0.1215 | 9 | 5 | 14 |
| 28 | *Camelina sativa* | 0.0445 | 117 | 0.1491 | 8 | 5 | 13 |
| 29 | *Camelina sativa* | 0.0458 | 176 | 0.1517 | 8 | 5 | 13 |
| 30 | *Camelina sativa* | 0.0405 | 31 | 0.1195 | 10 | 6 | 16 |
| 31 | *Camelina sativa* | 0.0467 | 183 | 0.1485 | 8 | 6 | 14 |
| 32 | *Camelina sativa* | 0.0405 | 110 | 0.1365 | 9 | 6 | 15 |
| 33 | *Camelina sativa* | 0.042 | 117 | 0.1539 | 8 | 7 | 15 |
| 34 | *Camelina sativa* | 0.04 | 90 | 0.1268 | 9 | 7 | 16 |
| 35 | *Camelina sativa* | 0.036 | 102 | 0.1113 | 9 | 6 | 15 |
| 36 | *Camelina sativa* | 0.037 | 56 | 0.1272 | 9 | 6 | 15 |
| 37 | *Camelina sativa* | 0.043 | 115 | 0.1305 | 8 | 6 | 14 |
| 38 | *Camelina sativa* | 0.0344 | 81 | 0.0998 | 10 | 5 | 15 |
| 39 | *Camelina sativa* | 0.0365 | 62 | 0.1119 | 9 | 5 | 14 |
| 40 | *Camelina sativa* | 0.0369 | 88 | 0.1211 | 9 | 6 | 15 |
| 41 | *Camelina sativa* | 0.0423 | 102 | 0.1473 | 8 | 5 | 13 |
